# A DNA Replication Stress-Based Prognostic Model for Lung Adenocarcinoma

**DOI:** 10.32607/actanaturae.25112

**Published:** 2023

**Authors:** S. Shi, G. Wen, C. Lei, J. Chang, X. Yin, X. Liu, S. Huang

**Affiliations:** Department of Cardiothoracic Surgery, The People’s Hospital of Dazu District, Chongqing, 402360 China; Department of Orthopedics, The People’s Hospital of Dazu District, Chongqing, 402360 China

**Keywords:** DNA replication stress, lung adenocarcinoma, prognostic model, immunotherapy response, anti- tumor drug prediction

## Abstract

Tumor cells endure continuous DNA replication stress, which opens the way to cancer development. Despite previous research, the prognostic implications of DNA replication stress on lung adenocarcinoma (LUAD) have yet to be investigated. Here, we aimed to investigate the potential of DNA replication stress-related genes (DNARSs) in predicting the prognosis of individuals with LUAD. Differentially expressed genes (DEGs) originated from the TCGA-LUAD dataset, and we constructed a 10-gene LUAD prognostic model based on DNARSs-related DEGs (DRSDs) using Cox regression analysis. The receiver operating characteristic (ROC) curve demonstrated excellent predictive capability for the LUAD prognostic model, while the Kaplan-Meier survival curve indicated a poorer prognosis in a high-risk (HR) group. Combined with clinical data, the Riskscore was found to be an independent predictor of LUAD prognosis. By incorporating Riskscore and clinical data, we developed a nomogram that demonstrated a capacity to predict overall survival and exhibited clinical utility, which was validated through the calibration curve, ROC curve, and decision curve analysis curve tests, confirming its effectiveness in prognostic evaluation. Immune analysis revealed that individuals belonging to the low-risk (LR) group exhibited a greater abundance of immune cell infiltration and higher levels of immune function. We calculated the immunopheno score and TIDE scores and tested them on the IMvigor210 and GSE78220 cohorts and found that individuals categorized in the LR group exhibited a higher likelihood of deriving therapeutic benefits from immunotherapy intervention. Additionally, we predicted that patients classified in the HR group would demonstrate enhanced sensitivity to Docetaxel using anti-tumor drugs. To summarize, we successfully developed and validated a prognostic model for LUAD by incorporating DNA replication stress as a key factor.

## INTRODUCTION

Lung cancer (LC) is a highly heterogeneous and lethal malignancy, representing a significant contributor to cancer incidence and mortality rates [1]. Lung adenocarcinoma (LUAD) stands as the predominant subtype of LC [2]. Surgery and radiation therapy offer hope for curing LUAD patients, while chemotherapy, targeted therapy, and immunotherapy can maximize the improvement of tumor prognosis. However, the prognosis for patients with LUAD still poses a significant challenge, with a relatively low long-term survival rate [3]. Parameters such as tumor size, TNM staging, and tumor grading cannot meet the demands of prognosis prediction and more precise treatment guidance, and finding new evaluation methods is a pressing need for precision medicine. The establishment of robust prognostic risk models holds the potential to significantly enhance our ability to forecast the prognosis of individuals diagnosed with LUAD.

The preservation of genome integrity heavily relies on the integrity and accuracy of DNA replication. However, the DNA replication process constantly faces challenges from various intrinsic and extrinsic stresses, including DNA damage and other factors, which can pose threats to overall genomic stability [4]. Various obstacles that delay, prevent, or terminate DNA replication are defined as DNA replication stress [5]. DNA replication stress activated by oncogene abnormalities is an important factor affecting cancer progression. On the one hand, it abets genomic instability, advancing cancer development. On the other hand, it retards cell proliferation and triggers anti-cancer defense mechanisms to induce cell apoptosis or senescence [6]. Tumor cells frequently exhibit a prominent characteristic of chronic replication stress, which arises from the persistent presence of replication stress sources due to impaired replication stress responses, diminished repair protein activity, and ongoing proliferation signal transduction. This chronic replication stress contributes significantly to the genomic instability and aberrant cell proliferation observed in tumor cells [7]. Previous studies have found that the DNA replication stress-related genes POLQ, PLK51, RAD6, CLASPIN, and CDC14 can predict the prognosis of early and mid-stage non-small cell LC (NSCLC) patients [8]. Additionally, DNA replication stress is an important mechanism for the chemotherapy and targeted therapy of LC. The integration of immunotherapy with these therapies represented a compelling strategy to augment the efficacy of LC treatment [9]. Therefore, the value of DNA replication stress-related genes (DNARSs) lies in their potential to be valuable prognostic markers and aid in predicting drug efficacy in the context of LUAD.

The proportion of immune cell infiltration in the tumor microenvironment (TME) affects cancer patient survival and the immunotherapy response [10, 11]. The expression levels of immune checkpoint inhibitors (ICIs) like cytotoxic T lymphocyte-associated protein 4 (CTLA4) and programmed cell death protein 1 (PD1)/ programmed cell death ligand 1 (PD-L1) are usually significantly increased in hypoxic malignant tumors, and ICIs are more effective for a small proportion of LC patients [12]. However, there are currently no tools available for forecasting the efficacy of immunotherapy in LUAD individuals.

We hereby used bioinformatics analysis to assess LUAD feature genes related to DNA replication stress and analyzed their roles in predicting the prognosis and drug efficacy for LUAD individuals.

## MATERIALS AND METHODS

**Data collection**

Gene expression datasets of LUAD with complete clinical data, including age, gender, tumor grade, and TNM staging, were provided by The Cancer Genome Atlas (TCGA, https://portal.gdc.cancer.gov/) and Gene Expression Omnibus (GEO, https://www.ncbi.nlm.nih. gov/) databases. The TCGA-LUAD dataset (539 cancer tissue samples and 59 normal tissue samples) was utilized as the training set, while the GSE26939 dataset (116 LUAD cancer tissue samples, platform number GPL9053) was used as the validation set.

Twenty-one DNA replication stress features were obtained from references, including 982 DNARSs (*Table 1*) [13, 14].

We collected the gene sequencing data of 119 tumor samples from individuals with urothelial cancer treated with atezolizumab (anti-PD-L1) from the IMvigor210 immune therapy cohort [15]. The GSE78220 dataset (platform number GPL11154) contained tumor samples from melanoma patients treated with anti-PD-1 therapy and was supplied by the GEO database [16].

**Differential analysis**

The R package “edgeR” [17] was used to conduct a differential analysis on LUAD tissue specimens and normal tissue specimens in the training set, and the differentially expressed genes (DEGs) of LUAD were selected along the criteria of standard FDR < 0.05 and |log(FC)| > 1. The intersection of DEGs and DNARSs was used to obtain the LUAD differential genes associated with DNA replication stress (DRSDs).

**Prognostic model construction and evaluation**

We first screened LUAD tumor patient specimens with a survival time greater than 30 days from the training set based on clinical data. Then, the univariate Cox regression analysis was tapped utilizing the R package “survival” (https://CRAN.R-project.org/ package=survival) to select the genes in DRSDs significantly associated with the overall survival (OS) of LUAD individuals. To mitigate the risk of overfitting in the statistical model, we employed the LASSO Cox analysis to identify a subset of feature genes from the larger pool of identified genes, utilizing the R packages “glmnet” [18] and “survival.” Feature genes were subjected to a multivariate Cox regression analysis to establish the LUAD prognostic model, using R packages “survival” and “survminer” (https://rdocumentation. org/packages/survminer/versions/0.4.9). The formula for calculating the Riskscores was

Riskscore = Σ Coefficient (gene) × Expressionvalue (gene).

Coefficient is the coefficient of the gene. Expressionvalue is the relative expression level of gene standardized by Z-score.

Riskscore was calculated for each LUAD patient sample in both the training and validation sets, and the samples were separated as high-risk (HR) and low-risk (LR) groups as per the median value. The distribution of Riskscore scores, patient survival status, and expression levels of feature factors in the two risk groups of LUAD patient specimens in the training set were analyzed. Kaplan-Meier survival curves were constructed utilizing the R package “survival” to compare the difference in the survival rates between the patients in the two groups. Receiver operating characteristic (ROC) curves were constructed using the R packages “timeROC” [19] and “survival” to calculate the area under the curve (AUC) and test the prognostic performance of the model.

**Independent prognostic analysis, nomogram construction, and evaluation**

The Riskscore from the training set was used as the single feature and combined with clinical data to perform univariate Cox and multivariate Cox regression analyses, evaluating the independent ability of the model to predict the patient survival chances. A LUAD prognostic nomogram was constructed using clinical factors and Riskscore, and a calibration curve was utilized to evaluate the disparity between the predicted event rate and the actual event rate. The R packages “rms” [20] and “survival” were used for this analysis. The ROC curves were depicted utilizing the R packages “timeROC” [19] and “survival” to evaluate the performance of the model in forecasting the prognosis of LUAD patients based on nomogram, Riskscore, age, gender, tumor grade, and TNM staging. The standardized net benefit of the nomogram was analyzed using the decision curve analysis (DCA).

**Tumor immune analysis**

Immune infiltration analysis was done utilizing the R packages “GSVA” [21] and “estimate” (https://RForge. R-project.org/projects/estimate/). The ssGSEA method was used to analyze immune cell infiltration and function in the HR and LR groups, and the expression of human leukocyte antigen (HLA)-related genes was evaluated. The differences between different risk groups were compared using the Wilcoxon test.

**Prediction of immunotherapy response**

To forecast the response of the HR and LR groups to immunotherapy, a series of studies were conducted. Immune checkpoints expression was analyzed in the two groups. The immunophenoscore (IPS) demonstrates high accuracy in predicting the response to anti-CTLA-4 and anti-PD-1 therapies, making it a valuable tool for determining the tumor’s likelihood of responding to ICI therapy. The IPS score of each patient was obtained from The Cancer Immunome Atlas (TCIA, https://tcia.at), and the differences in IPS scores between the two groups were compared. Tumor Immune Dysfunction and Exclusion (TIDE) can forecast the response to immunotherapy by simulating the main mechanisms of tumor immune escape. We employed TIDE score to predict the response of the two groups to ICI immunotherapy.

Furthermore, we used the Imvigor210 immune therapy cohort of individuals with urothelial cancer treated with the anti-PD-L1 inhibitor atezolizumab and the GSE78220 transcriptome dataset of melanoma individuals treated with anti-PD1 to test the effectiveness of the model in predicting the response to immunotherapy, including treatment efficacy and survival.

**Anti-tumor drug screening**

To identify potential targets and effective drugs, we used the CellMiner database (https://discover.nci.nih. gov/cellminer/) and R package “pRRophetic” (https:// github.com/paulgeeleher/pRRophetic/) to screen for anti-tumor drugs related to the IC_50_ of feature genes. Different drug IC_50_ values were predicted in the two groups, with lower IC_50_ values indicating a more effective cancer treatment [22].

## RESULTS

**Identification of DRSDs**

**Fig. 1:**
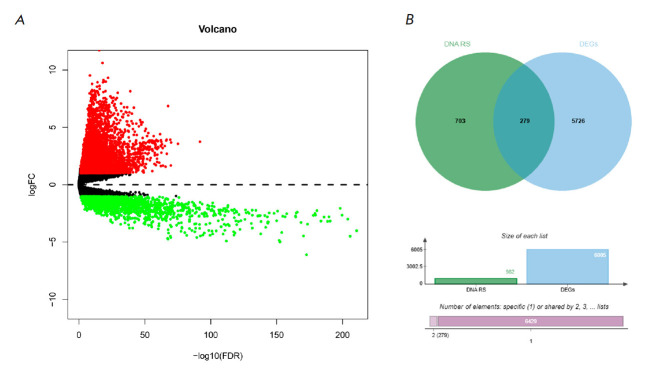
Screening of DRSDs. (*A*) Volcano plot of DEGs related to LUAD. (*B*) Venn diagram of the intersection between DEGs and DNARSs, corresponding to DRSDs

This study’s training set included expression data from 539 LUAD cancer tissue specimens and 59 normal tissue specimens. DEGs of the LUAD differential gene sets were obtained through a differential analysis, including 6,005 genes. Among the analyzed genes, we observed differential upregulation in 4,217 genes and differential downregulation in 1,788 genes (*Fig. 1A*, *Table 2*). Intersection of the DNARSs with 982 genes and DEGs was taken to obtain the Venn diagram of DRSDs, which contained 279 genes (*Fig. 1B*).

**Establishment of a prognostic model**

**Fig. 2:**
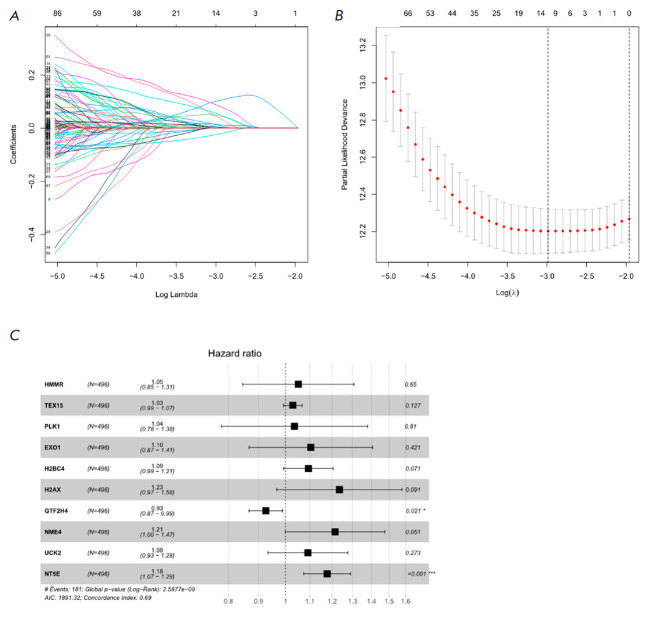
Construction of a LUAD prognostic model using DRSDs**.** (*A*) Cross-validation plot of the logarithmic (λ) sequence in the LASSO model, with the selection of the best parameter (lambda) indicated by the first black dotted line. (*B*) LASSO coefficient spectrum of 10 OS-related genes. (*C*) Forest plot of the multivariate Cox regression analysis based on the 10 feature genes in DRSDs

To develop robust risk features for clinical use, a series of Cox regression analyses were conducted. First, 163 genes that may affect OS were identified from the 279 genes in DRSDs through univariate Cox analysis. Then, 10 candidate genes were determined using LASSO regression (*Fig. 2A,B*). Multivariate Cox analysis showed that the coefficients of 10 feature genes were non-zero, with NT5E being a prognostic risk factor and GTF2H4 being a protective factor. The model was established ground on 10 genes (*Fig. 2C*). The 10-gene LUAD prognostic risk model based on DNA repair stress is shown below:

Riskscore = 0.05 × HMMR + 0.03 × TEX15 + 0.04 × PLK1 + 0.10 × EX01 + 0.09 × H2BC4 + 0.21 × H2AX - 0.08 × GTF2H4 + 0.19 × NME4 + 0.09 × UCK2 + 0.16 × NT5E

**Evaluation of the prognostic model**

**Fig. 3:**
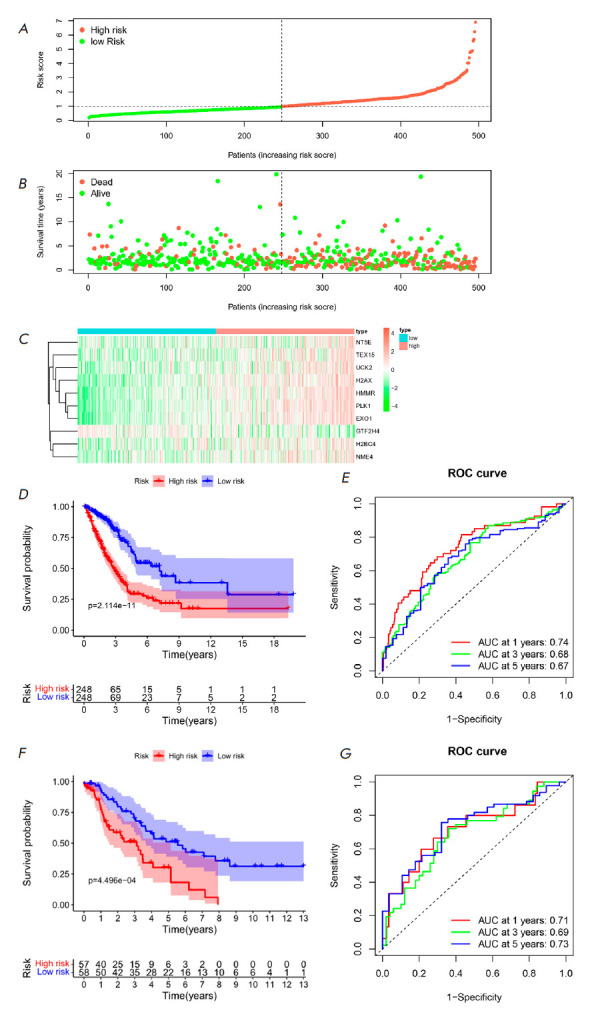
Performance evaluation of the prognostic model in predicting the prognosis risk of LUAD patients. (*A*) Distribution of Riskscore values in the TCGA training set, with the dotted line indicating the optimal threshold between the LR and HR groups. (*B*) Distribution of survival status in the TCGA training set, with the dotted line indicating the optimal threshold between the LR and HR groups. (*C*) Heatmap of the expression levels of the 10 feature genes in the TCGA training set. (*D*) Kaplan-Meier survival curve in the TCGA training set. (*E*) ROC curve in the TCGA training set. (*F*) Kaplan-Meier survival curve in the GEO validation set. (*G*) ROC curve in the GEO validation set

The Riskscore of LUAD samples in both the training and validation sets were computed by utilizing the LUAD prognostic risk model, and specimens were divided into HR and LR groups accordingly. The distribution of Riskscore values and survival status within the training set revealed that patients in the HR group exhibited a higher mortality rate (*Fig. 3A,B*). The heatmap of feature gene expression in the training set samples showed that all genes except GTF2H4 were highly expressed in the HR group (*Fig. 3C*). From the training set, we found that the survival rate of HR patients was lower (*P* < 0.05), indicating better overall prognosis for LR individuals (*Fig. 3D*). The ROC curve of the training set showed that the AUC values for 1-, 3-, and 5-year were between 0.67 and 0.74, indicating good sensitivity and specificity of the risk model (*Fig. 3E*). External validation of the validation set showed that patients in the HR group had a lower survival rate than those in the LR group (*P* < 0.05) (*Fig. 3F*). The ROC curve of the validation set showed the AUC values for 1-, 3-, and 5-year were between 0.69 and 0.73, proving that the risk model also did well in the validation set (*Fig. 3G*). In summary, the LUAD prognostic model based on exhibits high accuracy and reliability in predicting patient likelihood of survival.

**Independent prognostic analysis**

**Fig. 4:**
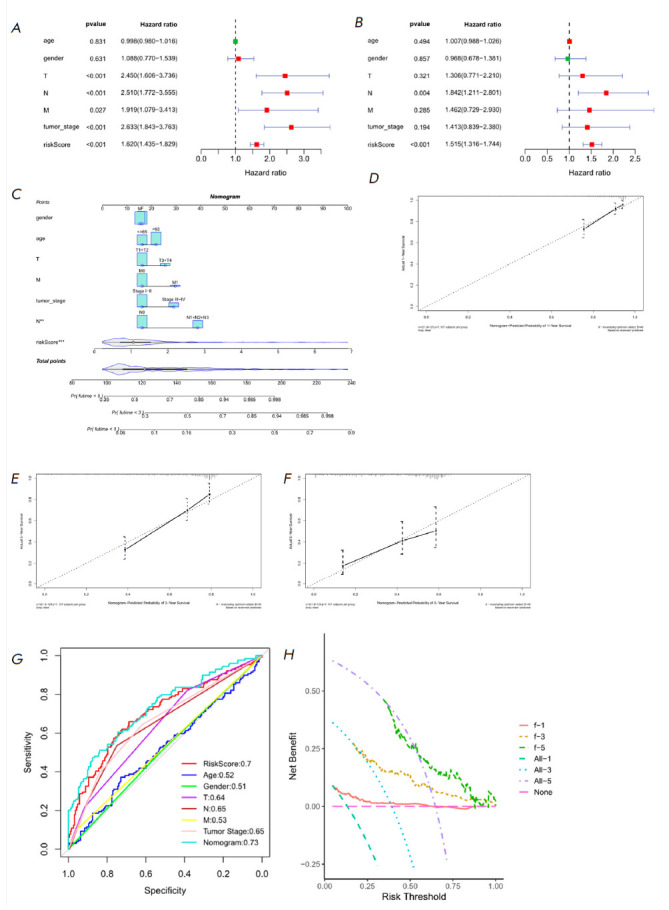
Independent prognosis analysis of Riskscore in LUAD patients in the TCGA training set. (*A*) Forest plot of the univariate Cox regression analysis combining Riskscore with clinical information. (*B*) Forest plot of the multivariate Cox regression analysis combining Riskscore and clinical information on LUAD patients. (*C*) Nomogram constructed by combining Riskscore and clinical information. (*D*), (*E*), and (*F*) Calibration curves for predicting the risk of 1-, 3-, and 5-year death, respectively. (*G*) Clinical features, Riskscore, and ROC curve used to diagnose Nomograms. (*H*) DCA curve for diagnosing Nomograms

To examine the independent impact of Riskscore on the survival of LUAD patients, we conducted both univariate and multivariate Cox analyses. These analyses involved incorporating the patients’ Riskscore along with other relevant clinical-pathological indicators. The findings revealed that Riskscore independently served as a prognostic factor for LUAD patients’ OS (*Fig. 4A,B*). Then, we combined Riskscore with prognostic clinical features to construct a nomogram for a more comprehensive prediction of patient chances of survival (*Fig. 4C*). According to the calibration curve, the nomogram predicted the OS of LUAD individuals at 1-, 3-, and 5-year with little difference from the ideal model (*Fig. 4D-F*). The ROC curve illustrated that the AUC values of Riskscore and the nomogram were 0.7 and 0.73, respectively, higher than those of other clinical factors, indicating good prognostic predictive ability (*Fig. 4G*). We analyzed the clinical net benefit of the nomogram via DCA curve analysis, which showed that the nomogram was of clinical utility in forecasting the prognosis of LUAD individuals (*Fig. 4H*). Therefore, the nomogram established here helped predict the survival probability of LUAD patients.

**Tumor immune cell infiltration**

**Fig. 5:**
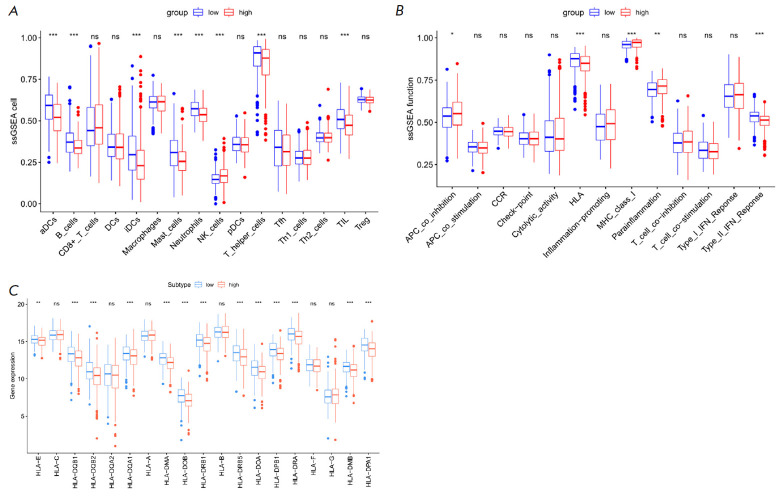
Analysis of immune cell infiltration and immune function between the high-risk and LR groups of LUAD patients using ssGSEA. (*A*) Analysis of immune cell infiltration. (*B*) Analysis of immune function. (*C*) Expression level analysis of HLA genes

Tumor immune cell infiltration is tightly linked to tumor progression [23]. By analyzing the immune cell infiltration and immune-related functional pathways between the two groups, we probed the disparities in the immune activity status between the two groups (*Fig. 5A,B*). The proportions of immune cell infiltration of dendritic cells (aDCs, iDCs), B_cells, Mast_cells, Neutrophils, T_helper_cells, and TIL were tellingly downregulated in the HR group (*P* < 0.05) (*Fig. 5A*). The immune-related pathway APC_co-inhibition was notably upregulated, while HLA and Type_II_IFN_ Response were significantly downregulated in the same group (*P* < 0.05) (*Fig. 5B*). In addition, most HLA genes were significantly downregulated in the same group (*P* < 0.05) (*Fig. 5C*). In summary, the proportion of immune cell infiltration in HR LUAD patients was lower compared to that in the LR group.

## DISCUSSION

**Prediction of immunotherapy response**

**Fig. 6:**
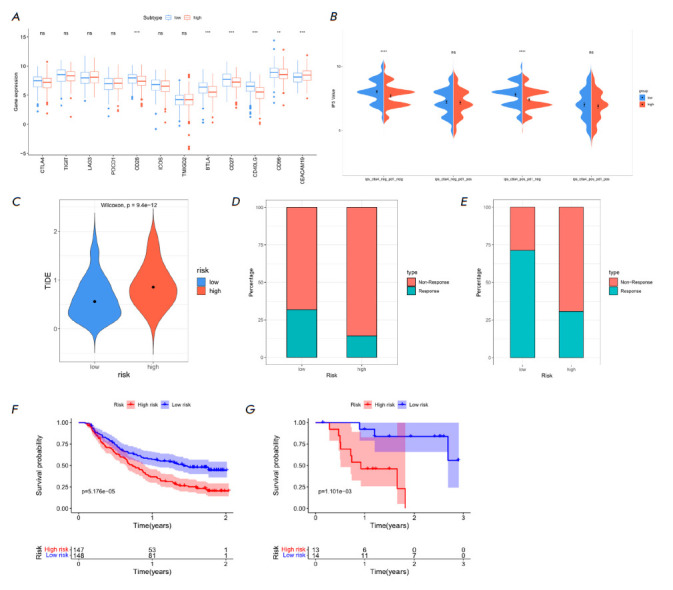
Analysis of the immunotherapy response in the HR and LR groups of LUAD patients. (*A*) Boxplot of immune checkpoint expression levels in the HR and LR groups of LUAD patients. (*B*) Violin plot of IPS scores in the HR and LR groups of LUAD patients. (*C*) Violin plot of TIDE scores in the HR and LR groups of LUAD patients. (*D*) ICI treatment response of the HR and LR groups of LUAD patients in the iMvigor210 cohort. (*E*) ICI treatment response of the HR and LR groups of LUAD patients in the GSE78220 cohort. (*F–G*) Kaplan-Meier survival curve of the HR and LR groups of LUAD patients in the iMvigor210 (*F*) and GSE78220 cohorts (*G*), respectively

The Riskscore of LUAD individuals is tightly linked to their immune function, suggesting that the HR and LR groups may have different responses to immunotherapy. Therefore, we further explored the ability of the prognostic model to predict the immunotherapy response of cancer individuals. Expression of most immune checkpoints was notably higher in the LR group, with significant differences (*P* < 0.05) (*Fig. 6A*). The IPS score indicated that individuals in the LR group exhibited a better response to CTLA-4 and anti-PD-1 treatment, denoting that LR LUAD individuals had stronger immunogenicity and were more likely to benefit from immune therapy (*P* < 0.05) (*Fig. 6B*). LR LUAD individuals with lower TIDE scores indicated a weaker inclination to evade the immune system and a stronger inclination to benefit from immune therapy, with significant differences (*P* < 0.05) (*Fig. 6C*). Since there is currently no transcriptome data on the response of LUAD individuals to ICI treatment, we used other cancer data to ascertain the performance of the model in predicting the immunotherapy response. Using the IMvigor210 and GSE78220 datasets to verify the response of the HR and LR groups, we found that the samples responsive to immunotherapy in the LR group were higher than those in the HR group (*Fig. 6D-E*), and that OS of the LR group was tellingly better than that of the HR group, showing a better survival trend (*Fig. 6F-G*). In summary, LR LUAD patients displayed a greater likelihood of responding to immunotherapy than HR patients and had a better prognosis.

**Prediction of potential anti-cancer drugs**

**Fig. 7:**
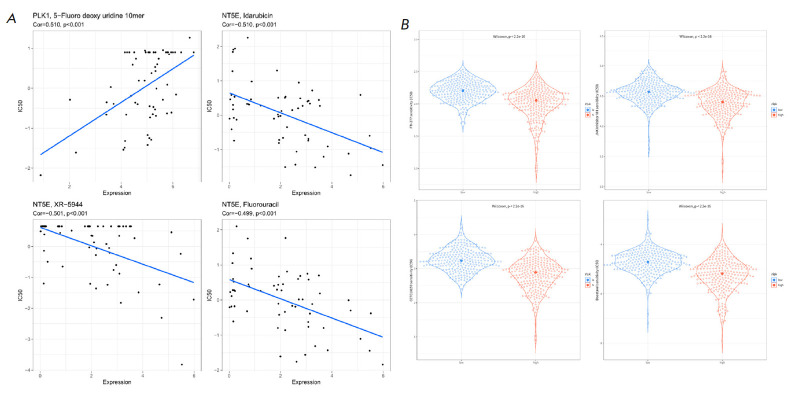
Prediction of the response of LUAD patients to anticancer drug inhibitors. (*A*) Correlation between the expression levels of feature genes in LUAD and the IC_50_ values of patients to drug inhibitors. (*B*) Prediction of the treatment response of LUAD patients to FTI-277, JNK Inhibitor VIII, CCT018159, and Docetaxel in the HR and LR groups of LUAD patients

To mine the response of LUAD patients to anti-cancer drug treatment, we dissected the linkage between the expression of prognostic feature genes and the IC_50_ values of drug antagonists, with results displaying a significant positive linkage between the expression of the PLK1 and IC_50_ value of 5-Fluoro deoxy uridine 10mer (cor = 0.510), while the expression level of NT5E showed a significant negative linkage with the IC_50_ values of Idarubicin (cor = -0.510), XR- 5944 (cor = -0.501), and Fluorouracil (cor = -0.499) (*Fig. 7A*). Furthermore, we investigated the association between the prognostic risk and drug sensitivity. The findings revealed that the HR group, characterized by a poor OS, exhibited heightened sensitivity to the drugs FTI-277, JNK Inhibitor VIII, CCT018159, and Docetaxel (*P* < 0.001) (*Fig. 7B*).

## CONCLUSION

Despite the availability of various treatments like surgery, radiotherapy, chemotherapy, targeted therapy, and immunotherapy, the mortality rate of LUAD remains high. DNA replication abnormalities are the main cause of genomic instability leading to tumor initiation and progression [24]. DNA replication stress not only affects the autonomous cell response of cancer patients, but also alters the cellular microenvironment, activates innate immune responses, and helps the organism to protect itself against proliferating damaged cells [25]. Here, we developed a LUAD prognosis model grounded in DNARSs. In the training and validation cohorts, our novel LUAD prognosis model showed a reliable prognostic prediction performance and can serve as an independent prognostic tool for LUAD patients. The nomogram grounded in the Riskscore and clinical factors exhibits reliability and accuracy in forecasting the survival probability of LUAD individuals. The LR group of LUAD patients is characterized by high anti-tumor immune cell infiltration and high immune activity status.

Based on the Cox regression analysis, we obtained ten DNA replication stress biomarkers that impact the prognosis for LUAD individuals, including HMMR, TEX15, PLK1, EXO1, H2BC4, H2AX, NME4, UCK2, NT5E, and GTF2H4. The expression levels of HMMR, TEX15, PLK1, EXO1, H2BC4, H2AX, NME4, UCK2, and NT5E increased with increase in Riskscore. High expression of HMMR fosters malignant behaviors in LUAD individuals [26]. PLK1 mediates the phosphorylation of SKA3 and enhances the stability of the SKA3 protein, thereby promoting the malignant progression of LC [27]. The high expression of the EXO1 gene is an independent risk factor for a poor prognosis of LUAD, and EXO1 can also predict the response to chemotherapy [28, 29, 30]. Phosphorylated γH2AX at Ser-139 is a cellular response to DNA double- strand breaks and DNA damage, which features in tumor cell apoptosis. Studies have reported that the expression of γH2AX can predict the efficacy of ICI treatment in LUAD [31, 32]. NME4 affects NSCLC by overcoming cell cycle arrest and enhancing cell proliferation [33]. UCK2 is a rate-limiting enzyme in the pyrimidine salvage synthesis pathway, which promotes LC cell proliferation and migration [34, 35]. The NT5E gene encodes CD73, which promotes LUAD proliferation and metastasis via the EGFR/AKT/ mTOR axis [36, 37]. Additionally, an upregulation in the expression of GTF2H4 results in a corresponding decrease in Riskscore. As research has revealed, a decreased expression of GTF2H4 is associated with a decreased DNA repair capacity. Genetic variations in GTF2H4 raise the risk of LC, and GTF2H4 is a potential predictor of clinical outcomes of platinum-based chemotherapy in NSCLC patients [38, 39]. Although the effects of TEX15 and H2BC4 on LUAD are unknown, the effects of other DNA replication stress biomarkers on the risk of LUAD patient prognosis echo the findings of this study.

ICI therapy has greatly improved the dilemma of cancer treatment, but the probability of a response to ICI therapy in LUAD individuals remains comparatively low, while the majority of cancer patients may not derive substantial benefits from immunotherapy drugs [40]. Compared with HR LUAD patients, LR individuals have higher IPS and significantly lower TIDE scores, indicating that LR LUAD individuals display a greater likelihood of benefiting from immunotherapy. In addition, based on prognostic genes and prognostic risk grouping, it is helpful to highlight the efficacy of chemotherapy drugs widely used in the clinical treatment of LUAD. Idarubicin is an anthracycline chemotherapy drug commonly used to treat malignant tumors like LC and leukemia [41]. Our results showed that LUAD patients with high expression of NT5E were more sensitive to Idarubicin. Docetaxel belongs to the taxane class of chemotherapy drugs and is utilized to treat non-small cell lung cancer. They stabilize microtubules by preventing depolymerization and cause cell death [42]. Research has shown that LUAD individuals with a high Riskscore are more sensitive to Docetaxel. In addition, research found that the DNA-targeted drugs XR5944 [43], HSP90, and DDX39B inhibitor CCT018159 [44], farnesyl transferase inhibitor FTI-277 [45], and the JNK inhibitor VIII [46] with potential cancer therapeutic effects are related to the risk score of LUAD individuals. In summary, the LUAD prognostic risk score calculated using DNA replication stress biomarkers had the potential to predict the drug treatment response.

In conclusion, we have established a new DRSDs feature with the potential to forecast the immunotherapy response of LUAD individuals. Undeniably, limitations exist. Although the prognostic value of the DRSDs feature we established has been fully validated in the TCGA and GEO cohorts, the retrospective and potential biases of this study still need attention. Secondly, this study only conducted analyses based on public databases, and it is necessary to attempt more* in vitro *and *in vivo *experiments to study the molecular mechanisms of DNARSs affecting LUAD. In addition, external clinical studies are needed to determine the potential estimation accuracy of the DRSDs feature for the prognosis of LUAD individuals who have not received or have received immunotherapy.
